# Efficacy and safety of sorafenib versus sunitinib as first-line treatment in patients with metastatic renal cell carcinoma: largest single-center retrospective analysis

**DOI:** 10.18632/oncotarget.7395

**Published:** 2016-02-15

**Authors:** Xinan Sheng, Zhihong Chi, Chuanliang Cui, Lu Si, Siming Li, Bixia Tang, Lili Mao, Bin Lian, Xuan Wang, Xieqiao Yan, Jun Guo

**Affiliations:** ^1^ Key Laboratory of Carcinogenesis and Translational Research (Ministry of Education/Beijing), Department of Renal Cancer and Melanoma, Peking University Cancer Hospital & Institute, Collaborative Innovation Center for Cancer Medicine, Beijing, China

**Keywords:** retrospective, sorafenib, sunitinib, efficacy, metastatic renal cell carcinoma

## Abstract

We conducted this largest, single-center, retrospective study to determine the efficacy of sorafenib versus sunitinib as first-line therapy for metastatic renal cell carcinoma (mRCC) in Chinese patients to validate the potential data on direct comparison of the efficacy of first-line treatment with sorafenib and sunitinib in the treatment of mRCC. From November 2006 to March 2015, we reviewed medical records from Peking University Cancer Hospital and found 169 patients receiving sorafenib (400 mg orally BID continuously in a 4-week cycle) and 165 patients receiving sunitinib (50 mg orally daily in a 6-week cycle; 4/2 schedule) as the first-line targeted therapy. Median follow-up was 23.0 months. In sorafenib and sunitinib groups, there is no significant difference in progression-free survival (PFS) (9.0 months [95%CI:8.00-12.00] vs 11.0 months [95%CI:9.00-14.00], respectively; *P*=0.6289) and overall survival (OS) (28.0 months [95%CI:24.00-34.00] vs 28.0 months [95% CI:19.00-33.00], respectively; *P*=0.979). Subgroup analysis based on Karnofsky performance status (KPS), pathological type, Memorial Sloan Kettering Cancer Center score, and metastasis was also conducted. Multivariate analysis revealed that sorafenib treated patients had superior efficacy in patients with a KPS of <90 and significantly better PFS (hazard ratio: 0.460 [95% CI:0.222-0.954]). Most common adverse events were hand-foot skin reaction and thrombocytopenia which were manageable. Overall, no significant differences were seen between sorafenib and sunitinib in the treatment of advanced renal cancer. However, fewer toxicities associated with sorafenib and superior efficacy in subgroups (non-clear cell carcinoma and KPS <90) indicates sorafenib as an effective first-line treatment agent in patients with mRCC.

## INTRODUCTION

The advent of novel targeted molecular therapies has completely changed the therapeutic landscape of metastatic renal cell carcinoma (mRCC). Because these therapies have better efficacy and tolerability than cytokine therapy and have drastically improved prognosis, these are now considered as the clinically relevant first- and second-line treatment approaches for patients with mRCC [1].

Currently, seven targeted agents have been approved for the treatment of mRCC, all of which showed efficacy and tolerable safety in large phase III clinical trials. Among them, six targeted agents are recommended for first-line treatment of mRCC patients with favorable and intermediate risk [2, 3]. Temsirolimus has been a preferred choice for poor prognosis patients. For the favorable and intermediate risk patients, selecting the first-line regimen for a particular patient has become a hard decision for clinicians [3]. There were two phase III trials compared the targeted agents in the first line setting, for example pazopanib versus sunitinib (COMPARZ study) and axitinib versus sorafenib for the first line treatment [4,5]. However, till date limited reported data is available on the direct comparison between the other targeted agents.

Both sorafenib and sunitinib are oral multikinase inhibitors that target tumor proliferation and angiogenesis by inhibiting VEGFRs, platelet-derived growth factor receptors (PDGFRs), FMS-like tyrosine kinase 3 (Flt-3), c-Kit protein, and RET receptor tyrosine kinases; however, they do not have identical kinase inhibition profiles [6–8]. The target site for both the drugs is VEGFR 1–3, platelet-derived growth factor receptor, and c-Kit. In addition, sorafenib inhibits intracellular serine/threonine kinases such as C-Raf, wild-type B-Raf, and mutant B-Raf. It also has a potent antiangiogenic and proapoptotic activity, thereby representing a marked antitumoral effect [3, 6–10].

Evidence-based medicine suggested sorafenib as the second-line treatment after cytokine therapy (category 1) and its use as the first-line agent is only in selected patients (category 2A) [11]. However, compared with Western patients with advanced or metastatic RCC, Chinese patients with the same disease respond better to sorafenib as first-line targeted therapy [7, 12]. Moreover, results from TIVO-1 trial suggested that sorafenib as a first-line mRCC therapy yielded progression-free survival (PFS) of 9.1 months [13]. It is noteworthy that sorafenib is widely recommended in the China as first-line therapy for mRCC because of its significant efficacy and safety profile [7, 14, 15].

Sunitinib demonstrated longer overall survival compared with IFN-alpha (26.4 v 21.8 months, respectively) plus improvement in response and PFS in the first-line treatment of patients with metastatic RCC in phase III clinical trials. The overall survival event profiles highlight an improved prognosis in patients with RCC indicating a better treatment modality in the era of targeted therapy [16].

Despite the established efficacy of both the drugs, to date, there exists only one retrospective analysis from South Korea which indicated that sorafenib has comparable efficacy to sunitinib in the treatment of mRCC patients and fewer and less severe toxicities; however, definite conclusion cannot be drawn owing to small sample size [17]. Furthermore, previous analyses have different limitations with different prognostic or predictive factors and clinical studies directly comparing these multikinase inhibitors are still lacking. Overall, there still remains a paucity of information to validate the potential data on direct comparison of the efficacy of first-line treatment with sorafenib and sunitinib in the treatment of mRCC.

Hence, we conducted this largest retrospective study to primarily evaluate the efficacy and safety of first-line targeted agents (sorafenib versus sunitinib) administered in Chinese patients with mRCC. We also determined the factors predictive of PFS/OS with sorafenib or sunitinib.

## RESULTS

### Patient demographics and clinical characteristics

A total of 334 consecutive patients with mRCC who received first-line VEGF TKI treatment were screened from November 2006 to March 2015. Of these patients, 169 received sorafenib and 165 received sunitinib. The baseline characteristics were relatively well balanced between both the groups. Overall, the median age of the patients was 55 years, and majority of the patients were men (75.22%). Most of the patients had clear cell carcinoma (82.69%). Most of the patients received nephrectomy (98.2%), 74 out of these patients suffered cytoreductive nephrectomy and 5 patients without cytoreductive nephrectomy. The patient baseline demographic and clinical characteristics are summarized in Table 1.

**Table 1:** Baseline characteristics

Item	Overall population, n (%) (N = 335)	Sorafenib, n (%) (n = 169)	Sunitinib, n (%) (n = 166)	*P* value
Median age (years)	55	54	55	0.3445
Sex				
Male	252 (75.22)	125 (73.96)	127 (76.51)	0.5901
Pathological type				
Clear cell carcinoma	277 (82.69)	136 (80.47)	141 (84.94)	
Non-clear cell carcinoma	58 (17.31)	33 (19.53)	25 (15.06)	
Subtypes				0.2800
Papillary carcinoma	22 (6.57)	11 (6.51)	11 (6.63)	
Sarcomatoid	20 (5.97)	14 (8.28)	6 (3.61)	
Collecting duct carcinoma	8 (2.39)	5 (2.96)	3 (1.81)	
Chromophobe carcinoma	1 (0.29)	0 (0.00)	1 (0.60)	
Medullary carcinoma	1 (0.30)	1 (0.59)	0 (0.00)	
Chromosome translocation carcinoma	4 (1.19)	1 (0.59)	3 (1.81)	
Not elsewhere classifiable	2 (0.60)	1 (0.60)	1 (0.60)	
MSKCC risk group				0.0341
N (missing)	326 (9)	162 (7)	164 (2)	
Favorable risk	123 (37.73)	56 (34.57)	67 (40.85)	
Intermediate risk	182 (55.83)	90 (55.55)	92 (56.10)	
Poor	21 (6.44)	16 (9.88)	5 (3.05)	
Heng score				0.0017
N (missing)	325 (10)	161 (8)	164 (2)	
Favorable risk	123 (37.85)	55 (34.16)	66 (41.46)	
Intermediate risk	169 (52.00)	80 (49.69)	89 (54.27)	
Poor risk	33 (10.15)	26 (16.15)	7 (4.27)	
Presence of bone metastasis				0.3650
Yes	119 (35.52)	64 (37.87)	55 (33.13)	
Presence of pulmonary metastasis				0.1943
Yes	252 (75.22)	122 (72.19)	130 (78.31)	
Number of metastatic organs				0.6428
1	115 (34.33)	56 (33.14)	59 (35.54)	
≥2	220 (65.67)	113 (66.86)	107 (64.46)	
Use of second-line therapy				0.1096
Yes	94 (28.06)	54 (31.95)	40 (24.10)	

MSKCC, Memorial Sloan Kettering Cancer Center

### Efficacy of sorafenib versus sunitinib

**Figure 1:**
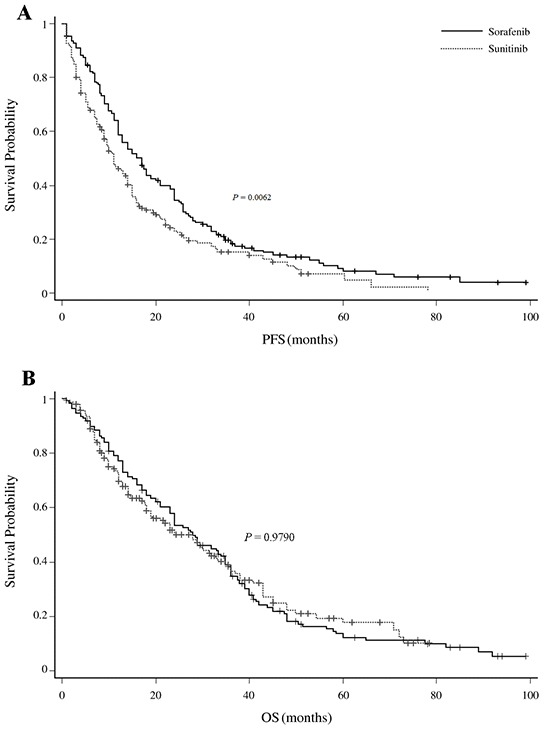
**A.** Comparison of PFS among sorafenib (all dose including escalated) versus sunitinib-treated patients. **B.** Comparison of OS among sorafenib versus sunitinib-treated patients.

At the date of censor, the median follow-up duration for all the patients was 23.0 months (95% CI: 20.5-25.5months). No significant differences in PFS were seen between the standard dose sorafenib and sunitinib groups (9.0 months [95% CI: 8.00-12.00] vs 11.0 months [95% CI: 9.00-14.00], respectively; *P* = 0.6289). However, an improved median PFS with prolonged survival was observed in patients receiving all dose sorafenib including escalated compared with those receiving sunitinib (17.0 months [95% CI: 13.00-19.00] vs 11.0 months [95% CI: 9.00-14.00], respectively; *P* = 0.0062; Figure 1A). No significant differences in OS were seen between the sorafenib and sunitinib groups (28.0 months ([95% CI: 24.00-34.00] vs 28.0 months [95% CI: 19.00-33.00], respectively; *P* = 0.979; Figure 1B).

**Figure 2:**
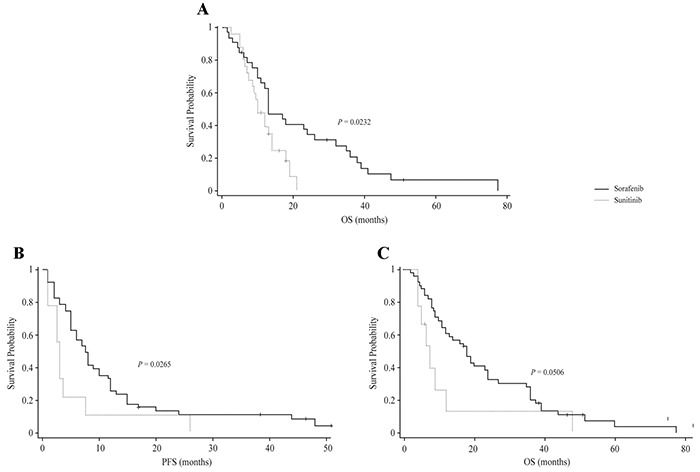
**A.** Subgroup Analysis: Comparison of OS among Sorafenib and Sunitinib Patients with Non-clear cell Carcinoma. **B.** Subgroup analysis: Comparison of PFS among Sorafenib and Sunitinib Patients with KPS of <90. **C.** Subgroup Analysis: Comparison of OS among sorafenib and sunitinib patients with KPS of < 90.

As per subgroup analysis, patients with non-clear cell renal cancer taking sorafenib showed the median PFS was numerically longer than with sunitinib, but the difference was not significant (8.50 months [95% CI: 5.00-10.00] vs 4.0 months [95% CI: 3.00-7.00]; *P* = 0.1221). There was significantly superior in OS (13.0 months [95% CI: 11.00-26.00] vs 10.0 months [95% CI: 7.50.00-14.00]; *P* = 0.0232; Figure 2A) compared with those taking sunitinib. However, the patient population is rather low in each arm to draw any definite conclusion.

On comparing the efficacy of sorafenib versus sunitinib based on Karnofsky performance status (KPS) score, patients with a KPS of < 90 taking sorafenib had a significantly superior PFS (7.50 months [95% CI: 5.00-10.00] vs 3.0 months [95% CI: 2.50-3.50]; *P* = 0.0265; Figure 2B) and OS (18.0 months [95% CI: 12.00-24.00] vs 7.5 months [95% CI: 5.00-12.00]; *P* = 0.0506; Figure 2C) compared with those taking sunitinib.

However, when the PFS and OS with sorafenib versus sunitinib were compared on the basis of whether the treatment interval exceeded 1 year for evaluation of time from diagnosis to systemic treatment administered, no significant differences were observed. Similarly, no significant differences were observed in PFS and OS between both the groups when evaluated among patients with MSKCC (favorable risk, intermediate risk and poor risk) and among patients with or without simple pulmonary metastasis.

### AEs and safety

Overall, all-grade and grade ≥3 AEs occurred in 331 (98.81%) and 174 (51.94%) patients, respectively. A significantly lower number of grade ≥3 AEs was seen in the sorafenib group than the sunitinib group (39.05% vs 65.06%; *P* < 0.0001). Moreover, the sorafenib group demonstrated significantly lower all-grade non-hematology AEs (94.67% vs 100.0%; *P* = 0.0074) and hematology AEs (49.11% vs 98.19%; *P* <0.0001), and grade ≥3 non-hematology AEs (25.44% vs 42.77%; *P* = 0.0008) and hematology AEs (18.34% vs 43.98%; *P* <0.0001) than the sunitinib group. Sunitinib group demonstrated a higher frequency of grade ≥3 hand-foot skin reaction (HFSR, 21.08% vs 14.20%; *P* = 0.0982) and thrombocytopenia (19.88% vs 1.18%; *P* <0.0001) than the sorafenib group. The patient distribution with respect to toxicity is outlined in Table 2.

**Table 2:** Adverse event in sorafenib and sunitinib groups

	Total population, n (%) (N = 335)		Sorafenib, n (%) (n = 169)		Sunitinib, n (%) (n = 166)		*P* value, n (%) (Nexavar vs Sutent)	
	All grade	Grade ≥3	All grade	Grade ≥3	All grade	Grade ≥3	All grade	Grade ≥3
All AE	331 (98.81)	174 (51.94)	165 (97.63)	66 (39.05)	166 (100.00)	108 (65.06)	0.1359	<0.0001
Non-hematology	326 (97.31)	114 (34.03)	160 (94.67)	43 (25.44)	166 (100.00)	71 (42.77)	0.0074	0.0008
HFSR	206 (61.49)	59 (17.61)	105 (62.13)	24 (14.20)	101 (60.84)	35 (21.08)	0.8088	0.0982
Diarrhea	183 (54.63)	15 (4.48)	90 (53.25)	6 (3.55)	93 (56.02)	9 (5.42)	0.6107	0.4076
Fatigue	150 (44.78)	22 (6.57)	62 (36.69)	9 (5.33)	88 (53.01)	13 (7.83)	0.0027	0.3546
Hypertension	128 (38.21)	19 (5.67)	59 (34.91)	6 (3.55)	69 (41.57)	13 (7.83)	0.2101	0.0903
Rash	117 (34.93)	10 (2.99)	66 (39.05)	2 (1.18)	51 (30.72)	8 (4.82)	0.1098	0.1022
Mucositis	112 (33.43)	18 (5.37)	40 (23.67)	10 (5.92)	72 (43.37)	8 (4.82)	0.0001	0.6559
Nausea	98 (29.25)	5 (1.49)	41 (24.26)	0 (0.00)	57 (34.34)	5 (3.01)	0.0427	0.0684
Anorexia	91 (27.16)	3 (0.90)	27 (15.98)	0 (0.00)	64 (38.55)	3 (1.81)	<0.0001	0.2398
Hypothyroidism	75 (22.39)	0 (0.00)	0 (0.00)	0 (0.00)	75 (45.18)	0 (0.00)	<0.0001	--
Dysgeusia	73 (21.79)	0 (0.00)	0 (0.00)	0 (0.00)	73 (43.98)	0 (0.00)	<0.0001	--
Vomit	63 (18.81)	7 (2.09)	28 (16.57)	0 (0.00)	35 (21.08)	7 (4.22)	0.2902	0.0206
Edema	53 (15.82)	2 (0.60)	0 (0.00)	0 (0.00)	53 (31.93)	2 (1.20)	<0.0001	0.2407
Hematology	246 (73.43)	104 (31.04)	83 (49.11)	31 (18.34)	163 (98.19)	73 (43.98)	<0.0001	<0.0001
Thrombocytopenia	126 (37.61)	35 (10.45)	27 (15.98)	2 (1.18)	99 (59.64)	33 (19.88)	<0.0001	<0.0001
Neutropenia	118 (35.22)	28 (8.36)	30 (17.75)	8 (4.73)	88 (53.01)	20 (12.05)	<0.0001	0.0156
Lymphopenia	108 (32.24)	33 (9.85)	40 (23.67)	17 (10.06)	68 (40.96)	16 (9.64)	0.0007	0.8972
Anemia	72 (21.49)	21 (6.27)	14 (8.28)	5 (2.96)	58 (34.94)	16 (9.64)	<0.0001	0.0117

HFSR, hand-foot skin reaction

### Response rates

In the sorafenib group, 18 patients (10.65%) achieved PR and 142 (84.02%) patients had SD, whereas in the sunitinib group, 2 patients (1.20%) achieved CR, 51 (30.72%) reported PR, 87 patients (52.42%) had SD and 26 patients (15.66%) demonstrated PD, (Table 3). Overall, the disease control rate (DCR = CR + PR + SD) was higher in sorafenib-treated patients than sunitinib-treated patients (94.67% vs 84.33%, respectively), and the ORRs (CR + PR) were 10.65% and 31.92% in sorafenib- and sunitinib-treated patients, respectively.

**Table 3:** Response rates in sorafenib and sunitinib groups

Best response	Total population, n (%)	Sorafenib, n (%)	Sunitinib, n (%)
N (missing)	335 (0)	169 (0)	166 (0)
CR	2 (0.60)	0 (0.00)	2 (1.20)
PR	69 (20.60)	18 (10.65)	51 (30.72)
SD	229 (68.35)	142 (84.02)	87 (52.42)
PD	35 (10.45)	9 (5.33)	26 (15.66)

CR, complete response; PR, partial response; PD; partial disease; SD, stable disease

### Prognostic factors

**Figure 3:**
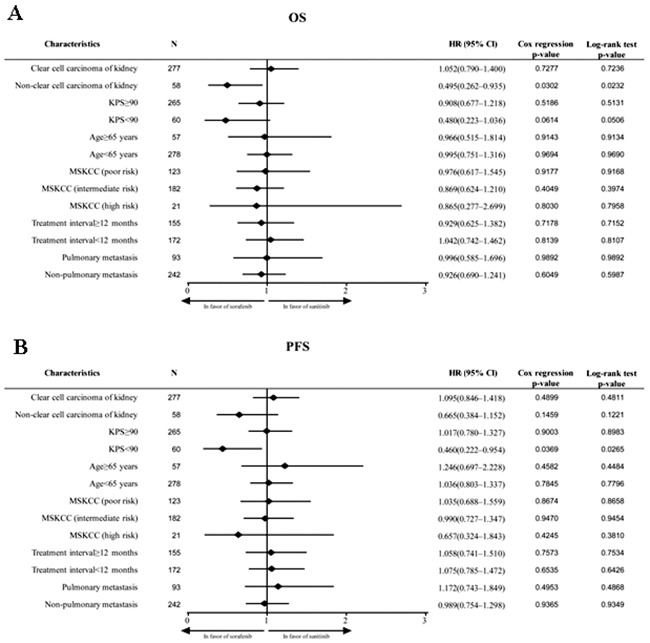
**A.** Forest plot for subgroup analysis of OS. **B.** Forest plot for subgroup analysis of PFS.

The prognostic factor analysis indicates that pathological type/treatment interval/number of metastatic organs/hemoglobin/LDH are the main factors affecting advanced renal cancer. Univariate and multivariate analyses revealed that prognostic factors such as non-clear cell carcinoma of the kidney, KPS (≥90 and <90), age (≥65 and <60 years), MSKCC (favorable risk, intermediate risk and poor risk), treatment interval (≥12 and <12 months), and non-pulmonary metastasis were associated with better OS in patients receiving sorafenib as compared to those receiving sunitinib. Moreover, sorafenib had a superior efficacy for OS in patients with non-clear cell carcinoma (HR: 0.495 [95% CI: 0.262-0.935]; *P* = 0.0302; Figure 3A). Multivariate analysis revealed that sorafenib had superior efficacy in patients with a KPS of <90, associated with significantly better PFS (HR: 0.460 [95% CI: 0.222-0.954]; *P* = 0.0369; Figure 3B). Overall, these results favored sorafenib as first-line targeted drug for the treatment of advanced RCC.

## DISCUSSION

In the current retrospective analysis, the results suggested that the efficacy of sorafenib is comparable to that of sunitinib in the first line patients with mRCC (PFS of 9.0 vs. 11.0 months and OS of 28.0 vs. 28.0 months, respectively). Moreover, adverse effects were significantly less frequent in the patients treated with sorafenib. To our knowledge, this is the largest clinical retrospective, comparative study to investigate the efficacy of first-line targeted therapy agents (sorafenib vs sunitinib) for mRCC.

Park et al conducted a retrospective analysis and concluded that the efficacy of sorafenib is comparable with that of sunitinib in 269 VEGF-TKI–naive patients with mRCC (sorafenib vs sunitinib: PFS, 8.6 vs 9.9 months; OS, 25.7 vs 22.6 months, respectively) [17]. The results of our analysis are in similar lines as compared with Park et al. however, it is noteworthy that our study has larger sample size and hence, a meaningful conclusion can be made out of it.

Oncologists believe PFS as a credible end point in oncology trials [18]. The phase III TARGET (Treatment Approach in Renal Cell Global Evaluation Trial) demonstrated that sorafenib improved PFS two-fold compared with placebo in 903 patients with clear cell mRCC who were refractory to prior cytokine therapy [9]. Based this study, sorafenib was approved by FDA and became the first targeted agent in the mRCC as the second-line treatment after cytokine therapy (category 1) [11]. However, in a randomized phase II trial of first-line sorafenib versus IFN-α, sorafenib resulted in a similar PFS to that of IFN-α (5.7months versus 5.6 months) [19]. Therefore, NCCN guideline recommended sorafenib as the first-line agent only in selected patients (category 2A) [11]. In the phase 3 trial comparing axitinib and sorafenib for mRCC patients with treatment-naïve, the median progression-free survival was 6.5 months in the sorafenib arm [4]. However, results from TIVO-1 trial suggested that sorafenib as a first-line mRCC therapy yielded PFS of 9.1 months which is longer than the median PFS from previous two studies [13]. This variability in results might be due to the prognostic differences among the patient population.

As reported previously, sorafenib had better clinical outcome in Asian mRCC patients [7, 12, 14]; moreover, several studies evaluated the efficacy of sorafenib in terms of PFS [12, 17, 20, 21]. A Japanese phase II trial of sorafenib in patients with mRCC who did not respond to cytokine treatment also demonstrated the efficacy of sorafenib with a DCR of 87.8%, a response rate of 14.7% and PFS of 32 weeks (95% CI, 25–40 weeks) [22]. In 2015, Guo et al demonstrated that sorafenib was efficacious in 131 patients with mRCC in Northeast China, with median PFS of 10.5 months, respectively, after a median follow-up of 16.9 months. The median PFS was 9.0 months in our study. The median PFS result was similar with the study in others Chinese patients [20]. In our study, the median PFS was 9.0months and similar with others Asian study.

In a randomized phase III trial comparing sunitinib with IFN-α, the median PFS was 11.0 months for the sunitinib arm and median overall survival of more than 2 years with sunitinib therapy [16]. Qin et al conducted a phase IV clinical trial in Mainland China evaluated the efficacy of sunitinib in patients with advanced RCC and reported DCR to be 76.7% with an objective response of 31.1% and a median PFS of 14.2 months [23]. As per the observations of our study the median PFS for the sunitinib treatment patients was 11.0 months and is similar to the previously reported data.

Our study results suggested that the efficacy of sorafenib was comparable with that of sunitinib in VEGF-TKI–naive patients with mRCC (9.0 months [95% CI: 8.00-12.00] vs 11.0 months [95% CI: 9.00-14.00], respectively; *P* = 0.6289). There was no difference between the two arms. As reported, median PFS was 3.6 months for patients escalated to sorafenib 600 mg twice daily in the phase II trial of first-line treatment with sorafenib versus interferon alfa-2a [19]. In our study, the median all dose sorafenib treatment time (included standard dose treatment plus escalated sorafenib time) was 17.0 months. Many clinical studies showed that RCC patients who tolerated the standard dose of sorafenib could benefit from the escalated dose of sorafenib after the disease progressed. For example, in one phase II clinical trial, the median PFS of RCC patients were prolonged 3.6 months after escalated dose sorafenib treatment [19]. An improved median PFS with prolonged survival was observed in patients receiving all dose sorafenib compared with those receiving sunitinib (*P* = 0.0062).

Results from a large, multicenter study conducted in 645 patients with anti-VEGF therapy-naive patients with mRCC treated with sorafenib, sunitinib, or bevacizumab as the first-line targeted therapy agents reported that although studies have reported variable PFS for first line VEGF TKI treatment in patients with mRCC, OS was not significantly different between patients who received sunitinib or sorafenib [24]. Our study demonstrated that the median OS was similar between patients who received sunitinib or sorafenib in the first line treatment. Moreover, higher number of patients had SD with sorafenib treatment versus sunitinib (84.02% vs 52.42%). Nevertheless, DCR rate was higher in sorafenib-treated patients compared with sunitinib-treated patients (94.67% vs 84.33%, respectively), whereas ORR (CR + PR) was lower with sorafenib (10.65%) than sunitinib (31.92%). Previous publications also have shown SD of 62-75% after sorafenib treatment. In phase III clinical studies (TARGET III), 74% patients had SD after sorafenib treatment [9]. In phase II study, 74.2% advanced kidney cancer patients had SD after first-line treatment with sorafenib [19]. In China, the clinical data showed a higher proportion of RCC patients who reached SD after sorafenib treatment, which was similar with previous reports.

Understanding and identifying prognostic factors is an essential tool for the development and evaluation of new treatments. Moreover, factors predictive for PFS/OS with targeting agents have not been fully assessed. Various pathologic markers such as prior nephrectomy, anemia, serum calcium level, lactate dehydrogenase, KPS, metastatic sites, blood neutrophil levels, and platelet levels hold a noticeable prognostic value in patients with mRCC undergoing targeted therapy [24, 25].

Therefore, a subgroup analysis was performed for the selection of better drug based on many prognostic factors such as age, sex, MSKCC score, pathological type, KPS score, treatment intervals, and metastatic lesions. In addition, prognostic factors for PFS and OS in Chinese patients with mRCC treated with sorafenib or sunitinib as first-line therapy were identified.

In our study, sorafenib showed significantly superior efficacy in some subgroups such as non-clear cell carcinoma patients (OS, 13 vs 10 months; *P* = 0.0232) and patients with a KPS of <90 (PFS, 7.5 vs 3.0 months; *P* = 0.0265; OS, 18 vs 7.5 months; *P* = 0.0506), which was of clinical benefit in this population. However, the patient population in the subgroup analysis is rather low and we suggest further studies to draw any definite conclusion on efficacy.

Selection of appropriate first-line therapy depends on the toxicity profile. Our study results demonstrated an acceptable safety profile in the Chinese population receiving sorafenib with mild-to-moderate toxicity, which was found to be consistent with the other studies [9, 14, 17].

A significantly lower grade ≥3 AEs were seen in the sorafenib group compared with the sunitinib (39.05% vs 65.06%; *P* <0.0001). All-grade and grade ≥3 non-hematology and hematology AEs were significantly lower in the sorafenib group compared with the sunitinib group. Patients with mRCC usually report HFSR as a common AE [9, 14, 17, 26], which was observed in our study as well, along with thrombocytopenia; however, the incidence was significantly lower in the sorafenib group than in the sunitinib group.

Taken together, this is the largest retrospective comparative study of sunitinib and sorafenib in patients with mRCC, a domain not much explored. Results are similar to the studies conducted previously, indicating that therapeutic profile of sorafenib is comparable with that of sunitinib with fewer and less severe toxicities associated with sorafenib.

Our analysis had few limitations. First, this is retrospective study and may affect the results of our analysis. Secondly, large-scale randomized clinical trials and sufficient duration are required to confirm our finding and to fully evaluate clinical outcomes. Consequently, the study population is more likely to be representative of the general oncology population and would provide information for optimizing the best treatment modality for the first treatment in the mRCC patients.

In conclusion, sorafenib may become an integral part of the standard care of first-line treatment in patients with mRCC, which has a profile comparable with that of sunitinib. Sorafenib had superior efficacy in some subgroups: patients with non-clear cell carcinoma and patients with a KPS of <90. Overall, this targeted agent was well tolerated with a manageable toxicity profile, even at higher dosages. It remains to be seen whether results of larger randomized and prospective studies will lead to a paradigm shift in treatment strategies for patients with mRCC.

## MATERIALS AND METHODS

### Study design and patient population

This was the largest, single-center, retrospective study conducted to determine the efficacy of sorafenib versus sunitinib as the first-line therapy for mRCC and to analyze survival. Patients were enrolled prospectively and their data were reviewed retrospectively. Medical records of all consecutive patients with mRCC who had been treated with sorafenib or sunitinib as the first-line targeted therapy at the Peking University Cancer Hospital were reviewed from November 2006 to March 2015.

Patients were eligible if they had histological confirmation of advanced mRCC; had a KPS of 70-100; had at least one or more measurable lesions; had adequate bone marrow, liver, and renal functions. Currently, there is no best biomarker and predictor factors for patient selection. So patient treatment were selected with sorafenib or sunitinib by the clinical experiences. Just for the patients with poor bone marrow function, the sorafenib was chosen as the first line treatment. Patients were excluded if they had unstable or severe cardiac disease and uncontrolled brain metastases.

The study protocol was approved by the local institutional review boards and ethics committees in accordance with the national and international guidelines and conformed to Declaration of Helsinki. Informed consent was signed and obtained from all the patients.

### Treatment

Sorafenib (400 mg) was administered orally twice daily continuously in a 4-week cycle until disease progression, unacceptable toxicities, or mortality. Sunitinib was administered at 50 mg orally daily in a 6-week cycle (4 weeks on, 2-week resting period—4/2 schedule) until disease progression, unacceptable AEs, or patient withdrawal.

We used the National Cancer Institute Common Terminology Criteria for Adverse Events v4.0 (NCI-CTC 4.0) to aid diagnosis and grading of treatment-related AEs, on the basis of which modifications in dose were decided [27]. For treatment-emergent toxicities of grade 3 or higher, therapy was withheld until resolution to grade 1 or lower. If the patient experienced grade 3 or 4 drug-related toxicity, second time, the dosage was decreased by one level. Patients who were initially intolerant to sunitinib 4/2 schedule, even after dose reductions, were then administered with the reduced dose for 2 consecutive weeks followed by 1-week resting period every 3 weeks (2/1 schedule). The subsequent dosing schedule depended on the treating physician's discretion.

For the second-line therapy, the dose of sorafenib was escalated to 600 mg in patients who tolerated the standard dose of sorafenib well. Once progressed, the dose of sorafenib was again increased to 800 mg BID. For the patients who tolerated the standard dose of sorafenib not well and who received sunitinib treatment in the first line treatment, they were eligible to take other TKI or mTORi in case of disease progression.

### Safety and response rate assessments

The primary end points of the study were PFS and OS. The secondary end points included treatment-related adverse events (AEs) and treatment response. Clinical, laboratory, and pathological data were collected and reviewed from patient medical records, and the most recent laboratory values were used before initiation of treatment. Pretreatment evaluation comprised a complete history and physical examination; complete blood count; liver, and renal function tests; CT scan of the chest, abdomen and pelvis; CT or MRI of the brain; and total body bone scan. Information on AEs and their severity was collected from the patient's medical records, and toxicity was analyzed and graded using the NCI-CTCAE v4.0 in all patients who received at least one dose of the drug (18). Radiological evaluation of the measurable tumor lesions was carried out every 2 cycles for the first year, and every 12 weeks thereafter in all the patients. Response was re-assessed according to RECIST (Response Evaluation Criteria in Solid Tumors) version 1.0 [28].

### Statistical analysis

All statistical analyses were performed using SAS version 9.1 (SAS Corporation, Cary, NC, USA) and Stata version 10 SE (StataCorp, College Station, TX, USA).

PFS was calculated from the start of first-line TKI treatment to the date of disease progression or death or the date of the last follow-up visit if the patients were still alive without progression. Median OS time was measured from the beginning of TKI until mortality or the date of the last follow-up visit if the patients were still alive. DCR was defined as the proportion of patients who achieved CR or PR, or SD. objective response rate (ORR) was defined as the proportion of patients who achieved CR and PR.

PFS and OS were analyzed by the Kaplan-Meier method and compared by the log-rank test. Clinical variables were evaluated by univariate and multivariate analyses using step-wise cox proportional hazard regression to evaluate associations with OS and PFS. All potential prognostic factors with a *P* value of ≤0.2 on univariate analyses were entered into the multivariate cox models. For PFS, OS, TTP, and time to first-line treatment failure, hazard ratios (HRs) and two-sided 95% confidence intervals (CIs) were derived from a cox proportional hazard model. ORR and DCR was assessed using Fisher exact test. Subgroup analysis and PFS/OS single factor analysis, including age, sex, pathological type, treatment interval, KPS score, MSKCC risk, Heng risk, presence of bone metastasis, presence of lung metastasis, number of metastatic organs (1 vs >1), and use of second-line therapy, were also assessed. Parameters such as age (>65 vs ≤65 years), sex, MSKCC score (poor vs intermediate risk), pathological type (clear cell vs non-clear cell carcinoma), KPS (<90 vs ≥90), treatment interval (<12 months vs ≥12 months), and metastatic lesions (simple vs non-simple pulmonary metastasis) provided guidance for drug selection. Safety was assessed in all patients receiving at least one dose of study drug and was summarized using descriptive statistics. All statistical analyses were two sided; a *P* value of <0.05 was considered significant.

## References

[1] Pal SK, Vogelzang NJ (2013). Sequential treatment strategies and combination therapy regimens in metastatic renal cell carcinoma. Clin Adv Hematol Oncol.

[2] Cho IC, Chung J (2012). Current status of targeted therapy for advanced renal cell carcinoma. Korean J Urol.

[3] Hutson TE (2011). Targeted therapies for the treatment of metastatic renal cell carcinoma: clinical evidence. Oncologist.

[4] Hutson TE, Lesovoy V, Al-Shukri S, Stus VP, Lipatov ON, Bair AH, Rosbrook B, Chen C, Kim S, Vogelzang NJ (2013). Axitinib versus sorafenib as first-line therapy in patients with metastatic renal-cell carcinoma: a randomised open-label phase 3 trial. Lancet Oncol.

[5] Motzer RJ, Hutson TE, Cella D, Reeves J, Hawkins R, Guo J, Nathan P, Staehler M, de Souza P, Merchan JR, Boleti E, Fife K, Jin J (2013). Pazopanib versus sunitinib in metastatic renal-cell carcinoma. N Engl J Med.

[6] Mendel DB, Laird AD, Xin X, Louie SG, Christensen JG, Li G, Schreck RE, Abrams TJ, Ngai TJ, Lee LB, Murray LJ, Carver J, Chan E (2003). In vivo antitumor activity of SU11248, a novel tyrosine kinase inhibitor targeting vascular endothelial growth factor and platelet-derived growth factor receptors: determination of a pharmacokinetic/pharmacodynamic relationship. Clin Cancer Res.

[7] Tan X, Liu Y, Hou J, Cao G (2015). Targeted therapies for renal cell carcinoma in Chinese patients: focus on everolimus. Onco Targets Ther.

[8] Wilhelm SM, Carter C, Tang L, Wilkie D, McNabola A, Rong H, Chen C, Zhang X, Vincent P, McHugh M, Cao Y, Shujath J, Gawlak S (2004). Progression and angiogenesis. Cancer Res.

[9] Escudier B, Eisen T, Stadler WM, Szczylik C, Oudard S, Siebels M, Negrier S, Chevreau C, Solska E, Desai AA, Rolland F, Demkow T, Hutson TE (2007). Sorafenib in advanced clear-cell renal-cell carcinoma. N Engl J Med.

[10] Zhao J, Zhu Y, Zhang C, Wang X, He H, Wang H, Wu Y, Zhou W, Shen Z (2013). Sorafenib or sunitinib as postoperative adjuvant therapy for Chinese patients with locally advanced clear cell renal cell carcinoma at high risk for disease recurrence. Urol Oncol.

[11] Molina AM, Motzer RJ (2011). Clinical practice guidelines for the treatment of metastatic renal cell carcinoma: today and tomorrow. Oncologist.

[12] Ye DW, Zhang HL (2014). Critical appraisal of sorafenib in the treatment of Chinese patients with renal cell carcinoma. Onco Targets Ther.

[13] Motzer RJ, Nosov D, Eisen T, Bondarenko I, Lesovoy V, Lipatov O, Tomczak P, Lyulko O, Alyasova A, Harza M, Kogan M, Alekseev BY, Sternberg CN (2013). Tivozanib versus sorafenib as initial targeted therapy for patients with metastatic renal cell carcinoma: results from a phase III trial. J Clin Oncol.

[14] Yang L, Shi L, Fu Q, Xiong H, Zhang M, Yu S (2012). Efficacy and safety of sorafenib in advanced renal cell carcinoma patients: Results from a long-term study. Oncol Lett.

[15] Ye DW, Shi GH (2012). Application of sunitinib for the treatment of advanced renal cell carcinoma in China: phase IV clinical trial. Zhonghua mi Niao Wai Ke Za Zhi.

[16] Motzer RJ, Hutson TE, Tomczak P, Michaelson MD, Bukowski RM, Oudard S, Negrier S, Szczylik C, Pili R, Bjarnason GA, Garcia-del-Muro X, Sosman JA, Solska E (2009). Overall survival and updated results for sunitinib compared with interferon alfa in patients with metastatic renal cell carcinoma. J Clin Oncol.

[17] Park SJ, Lee JL, Park I, Park K, Ahn Y, Ahn JH, Lee DH, Ahn S, Song C, Hong JH, Kim CS, Ahn H (2012). Comparative efficacy of sunitinib versus sorafenib as first-line treatment for patients with metastatic renal cell carcinoma. Chemotherapy.

[18] Johnson JR, Williams G, Pazdur R (2003). End points and United States Food and Drug Administration approval of oncology drugs. J Clin Oncol.

[19] Escudier B, Szczylik C, Hutson TE, Demkow T, Staehler M, Rolland F, Negrier S, Laferriere N, Scheuring UJ, Cella D, Shah S, Bukowski RM (2009). Randomized phase II trial of first-line treatment with sorafenib versus interferon Alfa-2a in patients with metastatic renal cell carcinoma. J Clin Oncol.

[20] Guo F, Han T, Liu Z, Song X, Zhang Q, Kong X, Li C, Li Z, Li C, Qu S, Zheng Z, Piao Y, Han Y, Xie X (2015). Prognostic analysis of Chinese patients with metastasis renal cell cancer receiving sorafenib: results from a multicenter long-term follow-up retrospective study. Onco Targets Ther.

[21] Zhang H, Dong B, Lu JJ, Yao X, Zhang S, Dai B, Shen Y, Zhu Y, Ye D, Huang Y (2009). Efficacy of sorafenib on metastatic renal cell carcinoma in Asian patients: results from a multicenter study. BMC Cancer.

[22] Akaza H, Tsukamoto T, Murai M, Nakajima K, Naito S (2007). Phase II study to investigate the efficacy, safety, and pharmacokinetics of sorafenib in Japanese patients with advanced renal cell carcinoma. Jpn J Clin Oncol.

[23] Qin S-K, Jin J, Guo J (2012). A phase IV multicenter study of the efficacy and safety of sunitinib as first-line therapy in Chinese patients with metastatic renal cell carcinoma. Ann Oncol.

[24] Heng DY, Xie W, Regan MM, Warren MA, Golshayan AR, Sahi C, Eigl BJ, Ruether JD, Cheng T, North S, Venner P, Knox JJ, Chi KN (2009). Prognostic factors for overall survival in patients with metastatic renal cell carcinoma treated with vascular endothelial growth factor-targeted agents: results from a large, multicenter study. J Clin Oncol.

[25] Patil S, Figlin RA, Hutson TE, Michaelson MD, Négrier S, Kim ST, Huang X, Motzer RJ (2011). Prognostic factors for progression-free and overall survival with sunitinib targeted therapy and with cytokine as first-line therapy in patients with metastatic renal cell carcinoma. Ann Oncol.

[26] Nakano K, Komatsu K, Kubo T, Natsui S, Nukui A, Kurokawa S, Kobayashi M, Morita T (2013). Hand-foot skin reaction is associated with the clinical outcome in patients with metastatic renal cell carcinoma treated with sorafenib. Jpn J Clin Oncol.

[27] Common Terminology Criteria for Adverse Events v4.0 (CTCAE) U S Department of health and human services 2010.

[28] Duffaud F, Therasse P (2000). [New guidelines to evaluate the response to treatment in solid tumors]. Bull Cancer.

